# Ectopic Gastric Mucosa in the Rectum: An Underrecognized Cause of Rectal Bleeding

**DOI:** 10.5152/tjg.2025.25133

**Published:** 2025-07-16

**Authors:** Fatih Kıvrakoğlu, Mustafa Ergin, Mehmet İbiş

**Affiliations:** 1 Adana City Training and Research Hospital, Adana, Türkiye; 2 Aksaray Training and Research Hospital, Aksaray, Türkiye; 3 Gazi University Faculty of Medicine, Ankara, Türkiye

Dear Editor,

Ectopic gastric mucosa (EGM) is an uncommon anomaly in the gastrointestinal tract, with most cases reported in the esophagus. However, its presence in the rectum is exceedingly rare, making it a diagnostic challenge. The authors would like to highlight a case of ectopic gastric mucosa in the rectum, which presented with intermittent rectal bleeding, to raise awareness about this under-recognized entity.

A 33-year-old male presented with fresh rectal bleeding persisting for a year. There were no associated symptoms of constipation, diarrhea, anal trauma, or a history of digital manipulation to assist defecation. During the patient’s first colonoscopy performed to investigate his symptoms, a 2 × 1 cm lesion was observed less than 1 cm from the anal verge, characterized by central depression and slightly elevated margins, consistent with the Paris Classification Type IIa. Narrow-band imaging revealed a regular microvascular and microsurface pattern (Figure 1). 

Histopathological examination confirmed ectopic gastric mucosa, characterized by positive MUC5AC staining, neutral mucin production, and the presence of *Helicobacter pylori* on histological analysis, ruling out dysplasia or malignant transformation. The patient was offered both endoscopic submucosal dissection (ESD) and surgical excision as treatment options, and he opted for surgery. He underwent surgical excision, and during a 24-month follow-up period, he remained asymptomatic with no evidence of recurrence. Written informed consent was obtained from the patient for the publication of this case report.

Heterotopic gastric mucosa is hypothesized to result from embryonic remnants failing to differentiate appropriately, leading to ectopic gastric tissue in unusual locations. Although the esophagus is the most common site, its presence in the rectum raises important clinical and diagnostic considerations. Misdiagnosis as hemorrhoids, inflammatory bowel disease, or colorectal neoplasia can delay appropriate intervention. Acid secretion from gastric-type epithelium may contribute to rectal ulceration and chronic bleeding, necessitating histopathological confirmation for accurate diagnosis. A limited number of case reports have described rectal EGM as a rare but underrecognized cause of rectal bleeding or polypoid lesions. Musa et al^1^ reported a case of rectal EGM incidentally detected during screening colonoscopy. Clarke et al^2^ described a case mimicking a high-risk anorectal neoplasm due to associated fundic gland polyps. Ruiz Marín et al^3^ and Ali et al^4^ presented cases of EGM with rectal bleeding requiring surgical excision. Kobori et al^5^ monitored a submucosal heterotopic gastric gland for 9 years and successfully treated it with endoscopic resection. These reports highlight the clinical heterogeneity and diagnostic challenges associated with this entity.

Endoscopic modalities such as narrow-band imaging and magnifying blue laser imaging provide valuable insights for early detection. In symptomatic cases, treatment options include ESD or surgical excision, depending on lesion size and patient preference. In our case, surgical resection provided definitive resolution without recurrence.

It is believed that ectopic gastric mucosa should be considered in cases of unexplained rectal bleeding, especially when conventional causes are ruled out. Increased awareness among gastroenterologists can lead to earlier diagnosis and appropriate management. Further studies are needed to explore the natural history, malignant potential, and optimal treatment strategies for this rare entity.

## Figures and Tables

**Figure 1.**
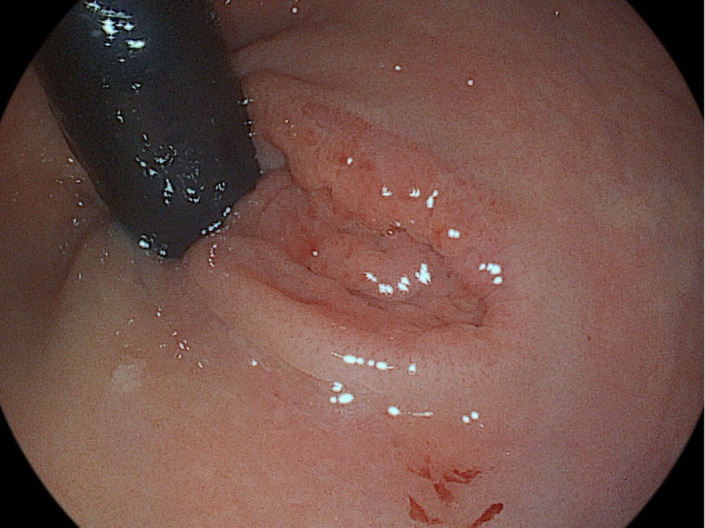
Endoscopic view of ectopic gastric mucosa in rectum.

## Data Availability

The data that support the findings of this study are available upon request from the corresponding author.
